# A Time-Encoded Technique for fibre-based hyperspectral broadband stimulated Raman microscopy

**DOI:** 10.1038/ncomms7784

**Published:** 2015-04-17

**Authors:** Sebastian Karpf, Matthias Eibl, Wolfgang Wieser, Thomas Klein, Robert Huber

**Affiliations:** 1 Lehrstuhl für BioMolekulare Optik, Fakultät für Physik, Ludwig-Maximilians-Universität München, Oettingenstreet 67, 80538 Munich, Germany; 2 Institut für Biomedizinische Optik, Universität zu Lübeck, Peter-Monnik-Weg 4, 23562 Lübeck, Germany

## Abstract

Raman sensing and microscopy are among the most specific optical technologies to identify the chemical compounds of unknown samples, and to enable label-free biomedical imaging. Here we present a method for stimulated Raman scattering spectroscopy and imaging with a time-encoded (TICO) Raman concept. We use continuous wave, rapidly wavelength-swept probe lasers and combine them with a short-duty-cycle actively modulated pump laser. Hence, we achieve high stimulated Raman gain signal levels, while still benefitting from the narrow linewidth and low noise of continuous wave operation. Our all-fibre TICO-Raman setup uses a Fourier domain mode-locked laser source to achieve a unique combination of high speed, broad spectral coverage (750–3,150 cm^−1^) and high resolution (0.5 cm^−1^). The Raman information is directly encoded and acquired in time. We demonstrate quantitative chemical analysis of a solvent mixture and hyperspectral Raman microscopy with molecular contrast of plant cells.

## Abstract (web summary)

Raman microscopes suffer from the compromise between speed and spectral information and are often unsuited for fibre beam delivery. Karpf *et al.* overcome these limitations using continuous-wave rapidly wavelength-swept probe lasers and a short-duty-cycle actively modulated pump laser in an all-fibre setup.

## Graphical abstract

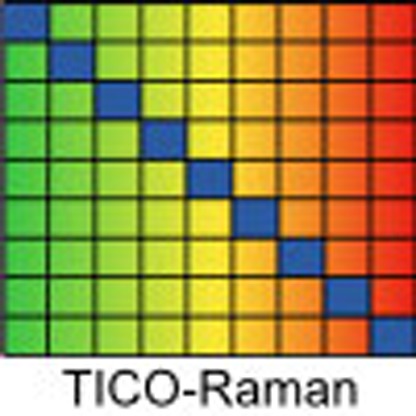

Raman spectroscopy is an optical technology directly sensitive to molecular vibrations. Therefore, it can provide a wealth of information about the chemical composition of a sample. In biomedical applications, it enables label-free molecular imaging *in vivo*. Being an optical technique, it can combine chemical contrast with high spatial resolution on a micron scale. Raman microscopy has great potential for diverse applications such as tumour detection^1^, drug delivery studies^2^,^3^ and biofuel process monitoring^4^,^5^.

Traditional linear Raman scattering suffers from inherently low signal, limiting its application mainly to non-imaging spectroscopy. The fundamental problem of the low signal can be overcome by nonlinear techniques, which can enhance the Raman intensity by many orders of magnitude.

The most popular nonlinear Raman techniques are coherent anti-stokes Raman scattering and stimulated Raman scattering (SRS). With coherent anti-stokes Raman scattering (CARS), high signal-to-noise ratios have been achieved^6^ and hyperspectral microscopy using a pulsed, stepwise wavelength-tuned laser has been demonstrated^7^. SRS systems using only one spectral position have pushed the speed up to video-rate^8^,^9^. To achieve hyperspectral imaging, multi-colour setups were developed^2^,^9^,^10^,^11^,^12^. Recent efforts concentrate on fibre delivery^13^,^14^,^15^,^16^ for future endoscopic applications^17^, and cost effective continuous wave (CW) systems^18^,^19^,^20^. However, the demand for a flexible, broadband, high-resolution Raman imaging system based on fibre light sources is still unmet^21^.

Here we present an SRS technique based on fastest wavelength-swept CW probe lasers. With the well-defined time characteristic of the wavelength sweep of this laser, the spectral Raman information is encoded and acquired in time. We call this approach time-encoded Raman (TICO-Raman). This technique can meet all the demands mentioned above; it is inherently low cost, readily compatible with endoscopes and has full spectroscopic capabilities. As an application, we show the acquisition of high-resolution SRS spectroscopy and hyperspectral Raman microscopy with this TICO-Raman system.

## Results

### TICO-Raman concept

**Figure 1:**
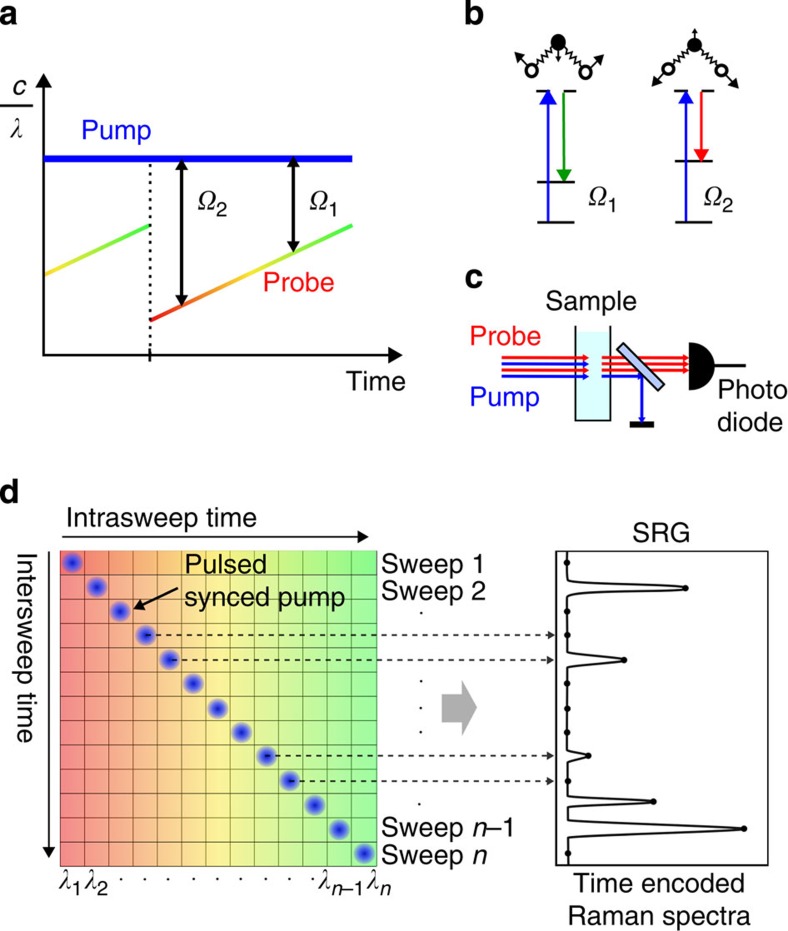
Concept and implementation of TICO-Raman. The concept: (**a**) a fixed wavelength pump and a swept wavelength probe laser scan molecular vibrations by changing the difference in photon energy. The probe laser experiences SRG when the energy difference matches Raman transitions (Ω_1_, Ω_2_). (**b**) Upon Raman scattering, pump photons are converted to probe photons and the loss in photon energy is transferred to molecular vibrations or rotations. (**c**) The SRG is measured as a signal change of the probe laser with a photodetector. Actual implementation: (**d**) the CW pump laser is modulated to nanosecond pulses, which are synchronized to the CW probe laser. The probe laser sweeps the wavelength over time (intrasweep time, rainbow coloured). TICO-Raman spectra are generated by positioning the pump pulses in time at probe wavelengths *λ*_1_,…,*λ*_n_ over consecutive sweeps 1,…,*n*. By direct time-resolved sampling of the probe laser intensity, *n* SRG measurement points are acquired. With the known time-to-wavenumber characteristic, the *n* values are mapped to a Raman spectrum.

The TICO-Raman concept is sketched in Fig. 1a–c. Two laser light sources, a Raman pump and a Raman probe laser, are focused onto the sample. A photodiode detects the probe power behind the sample (Fig. 1c). The pump laser operates at a fixed wavelength, whereas the probe light is repeatedly swept in wavelength over time (Fig. 1a). Therefore, the photon energy difference between the two lasers is changed periodically. Every time the difference matches a Raman transition of a sample molecule (Fig. 1b), pump photons will be converted to probe photons by SRS (Fig. 1c). Hence, the intensity of the probe laser at that specific spectral position gets increased by stimulated Raman gain (SRG). With the known time to wavelength encoding of the probe laser sweep, the SRG signals can be mapped to a Raman spectrum (see also Supplementary Fig. 3).

### TICO-Raman experimental implementation

In our actual implementation (Fig. 1d), we use a slightly different setup in order to combine the great potential of CW-SRS^18^,^19^,^20^ with the advantage of a pulsed pump laser with high-peak intensity. As the SRG scales linearly with the instantaneous pump power, we modulate the pump laser to low-duty cycles in order to achieve high-peak power levels at low average power. To generate Raman spectra, the timing of the pump pulses is successively increased with respect to the sweeps of the probe laser and the induced SRG signals are mapped to a spectrum (Fig. 1d). As part of this effort, we developed a pump laser that can generate three different wavelengths, which can be switched electronically. The pump laser is used in conjunction with two wavelength swept Fourier domain mode locked (FDML) probe lasers, each FDML laser having different emission spectra. This setup can potentially yield gapless coverage of Raman transitions from 250 cm^−1^ to 3,150 cm^−1^.

**Figure 2:**
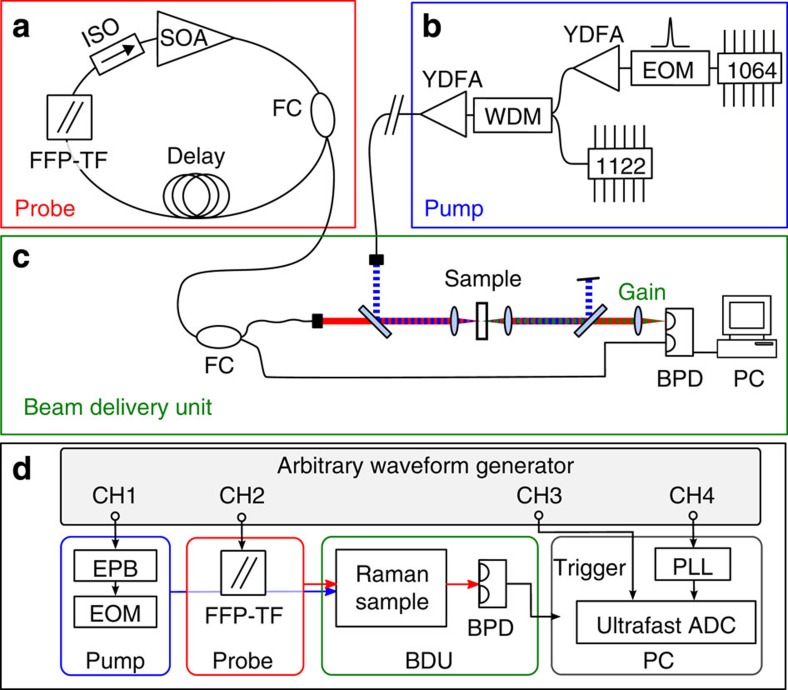
Setup of the TICO-Raman system. (**a**) The fibre based, wavelength-swept FDML probe laser^25^. FC, fibre coupler; FFP-TF, fibre Fabry-Pérot tuneable filter; ISO, optical isolator; SOA, semiconductor optical amplifier. (**b**) The homebuilt fibre-based pump laser is digitally synchronized to the FDML. EOM, electro-optic modulator; WDM, wavelength division multiplexer; YDFA, ytterbium-doped fibre amplifier. (**c**) The lasers are combined in the beam delivery unit and focused onto the sample. The SRG signal is detected after subtraction of the offset by a differential balanced photodetector (BPD). (**d**) Digital synchronization is employed by an inter-channel locked arbitrary waveform generator driving the whole TICO system. The SRG signals are directly sampled at 1.6 GS s^−1^ with a fast analogue-to-digital converter card and processed on a personal computer (PC).

The homebuilt fibre-based pump laser (Fig. 2b) combines the concept of a fibre-based master oscillator power amplifier (MOPA)^22^ with a wavelength shifter in the delivery glass fibre^23^,^24^. This allows to electronically switch the pump light wavelength from 1,064 nm to 1,122 nm or 1,185 nm (see also Supplementary Figs 1 and 2). Peak power, emission wavelength, duty cycle, repetition rate, pulse duration and pulse pattern can be dynamically programmed. This pump source is fully compatible with fibre endoscopes, as self-phase modulation—the main nonlinear effect causing pulse break up in fibres—is reduced by about three orders of magnitude by the use of nanosecond pulses instead of commonly used picoseconds pulses.

The fibre-based FDML probe laser (Fig. 2a) is a periodically wavelength-swept ring laser^25^. It uses a kilometre-long fibre to optically store the wavelength sweep, making it a very low noise, repetitive wavelength-swept CW light source.

FDML lasers achieve 150 nm wavelength sweep spans in the extended near infrared at high sweep repetition rates up to several MHz^26^. These sources are robust and are used for *in vivo* biomedical imaging in optical coherence tomography (OCT)^26^, including for applications in high-speed three-dimensional endoscopic OCT^27^. Like the pump laser, FDML probe lasers are highly flexible. Wavelength sweep range and centre wavelength can be freely reconfigured. The pump laser is electronically synchronized to the swept probe laser (Fig. 2d).

The intensity changes of the probe laser light due to SRG are small compared with the total intensity of the probe laser. Therefore, we apply a balanced detection scheme (Fig. 2c) to remove the probe light offset and, furthermore, eliminate laser noise. After this analogue balancing step, a second, digital balancing scheme additionally removes artefacts, such as signals arising from acoustic waves, thermal lensing and partial interference^28^. Together with the inherently low-noise FDML laser we achieve fundamental shot noise-limited detection sensitivity.

### TICO Raman spectroscopy

We investigated the advantages of the TICO-Raman system for two main applications: Raman spectroscopy and Raman microscopy. First, the spectroscopy performance is demonstrated.

**Figure 3:**
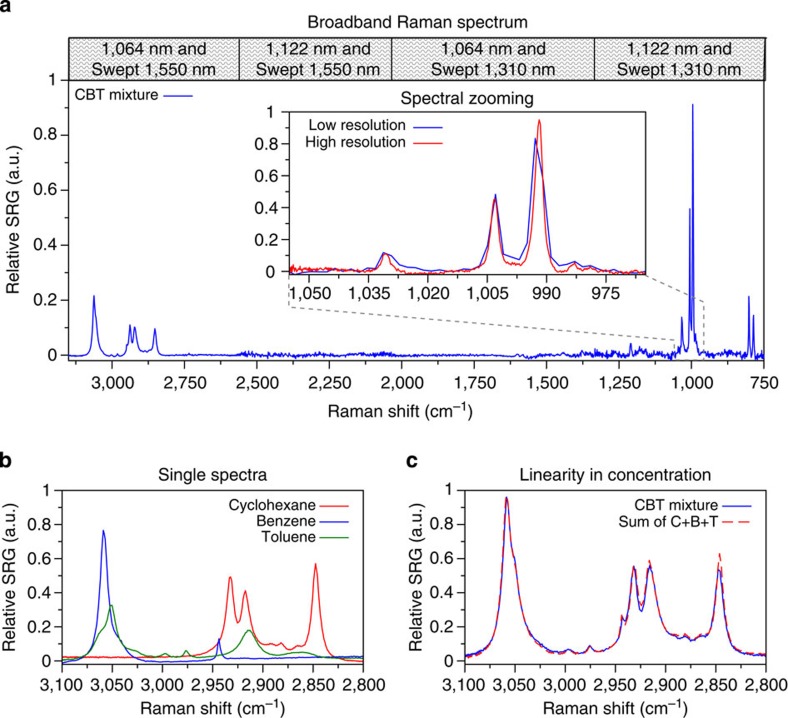
Quantitative chemical sensing. Here we show results for sensing of a mixture of cyclohexane, benzene and toluene (CBT, 1:1:1 ratio). (**a**) Broadband TICO-Raman survey spectrum from 750 to 3,150 cm^−1^ with a resolution of 3 cm^−1^. The spectrum has four sections acquired with two pump wavelengths and two FDML lasers. The inset shows the scope of dynamical spectral zooming. The sweep span of the swept laser is reduced, improving the spectral resolution to 0.5 cm^−1^. The good linearity of the TICO-Raman signal enables quantitative determination of different compounds. (**b**) Spectra of the individual liquids. (**c**) The spectrum of the chemical mixture (blue) and the mathematical sum of the single spectra (red, dotted) match very well.

The spectrum of a mixture of benzene, toluene and cyclohexane (Fig. 3a) has broadband coverage from 750 to 3,150 cm^−1^, a high resolution of better than 3 cm^−1^ and 1,565 spectral points. Four spectral blocks (coverage Fig. 3a top) were averaged 1,000 times and merged (for details see Supplementary Methods). The intensities were normalized by pump and probe power levels. The TICO-Raman spectra are in very good agreement with spontaneous Raman spectra (Supplementary Fig. 7).

Another unique feature of TICO-Raman is the possibility of dynamically zooming into a spectral region of interest by reducing the span of the probe laser. The effect of increased spectral resolution is shown in Fig. 3a, where the resolution improved from 3 cm^−1^ down to 0.5 cm^−1^. The two narrowband, neighbouring Raman peaks of toluene (1,005 cm^−1^) and benzene (992 cm^−1^) are better resolved after zooming in (see also Supplementary Fig. 4). It should be noted that the resolution in TICO-Raman is not limited by a spectrometer, but by the linewidths of the lasers. Owing to the pulse durations of nanoseconds, resolutions of GHz (∼0.007 cm^−1^) or better should be obtainable in the future^20^.

Another advantage of TICO-Raman, as it is an SRS technique, is the signal linearity in sample concentration. The left box in Fig. 3b shows three individual TICO-Raman spectra of cyclohexane, benzene and toluene. Figure 3b (right) shows a spectrum of the chemical mixture versus a spectrum calculated as weighted sum of the individual spectra. Both spectra agree very well, showing that TICO-Raman enables not only qualitative identification of the constituents of the examined sample but also their quantitative ratio.

### Hyperspectral TICO Raman microscopy

**Figure 4:**
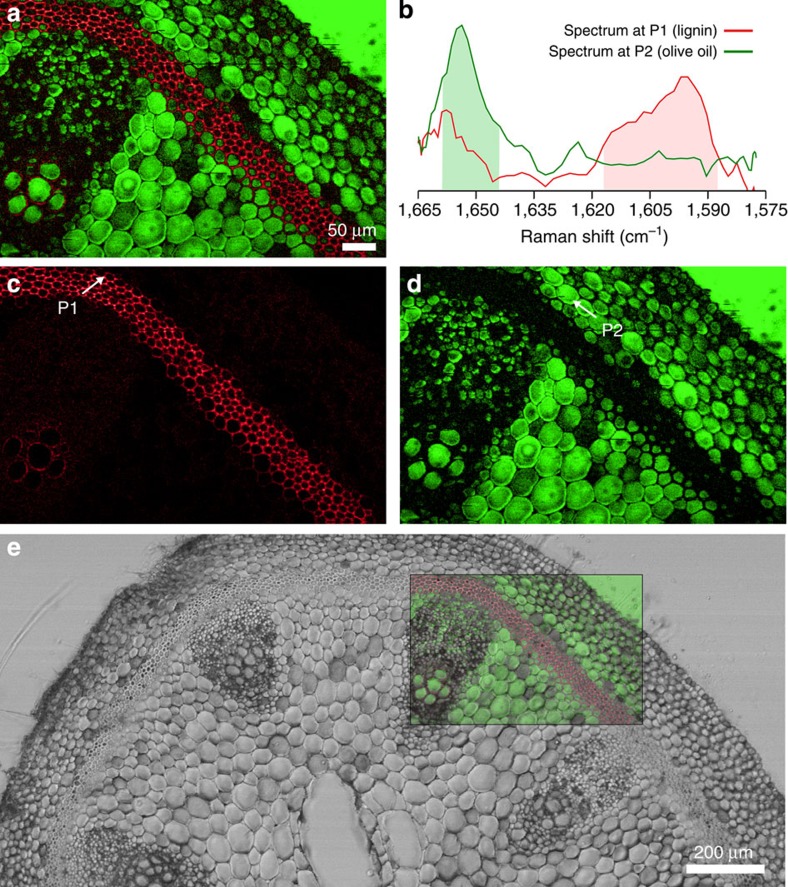
Hyperspectral TICO-Raman images of a slice of *Geranium phaeum* stem in olive oil. (**a**) Image with molecular colour code with lignin in red and olive oil in green. (**b**) Spectra at two pixels P1 and P2 and integration intervals used for colour coding (see also Supplementary Fig. 6). (**c**,**d**) High signal-to-noise images of the two-colour channels. (**e**) Morphological overlay of the molecular contrast image from the probe laser with the high-definition transmission microscopy image from the pump laser of the same setup.

As a second application of the TICO-Raman system, we performed hyperspectral SRS microscopy on plant cells by translating the sample. Figure 4 shows a slice of *Geranium phaeum* stem immersed in olive oil with molecular contrast. We chose this sample as a model because the lignin distribution in plants is highly relevant for research on biomass-to-biofuel conversion, as it is a key factor in the recalcitrance process^4^,^5^. Olive oil was chosen as a representative of the group of lipid molecules.

At each sample location, a 64-point spectrum from 1,575 to 1,665 cm^−1^ was acquired. The power on the sample was 3.3 mW for the probe laser and 480 mW for the pump (720 W instantaneous power). These power levels did not induce any damage on the sample. This may be due to the longer wavelength, as it has been shown that at 1,050 nm biological samples can handle ten times more power than the commonly applied 800 nm (ref. 29).

Usually, nonlinear imaging is performed in the 3,000 cm^−1^ region because of high signal levels, but the more specific region below 2,000 cm^−1^ would be preferred^4^.

We demonstrate the molecular distinction of lignin and olive oil around ∼1,600 cm^−1^ in the fingerprint region. The narrowband vibrations are only 60 cm^−1^ apart but clearly separable (Fig. 4b). Figure 4a shows the hyperspectral TICO-Raman image with lignin (red) and olive oil (green). The data were acquired at a speed of 415,000 spectral points per second (equivalent to 2.5 μs integration time). A total of 6,400 spectra are acquired per second (equivalent to 157 μs per spectrum; see also Supplementary Fig. 9). To directly assess the capabilities of our system to acquire high-quality spectra, we did not apply principle component analysis for image generation and spectral deconvolution. For sufficient signal-to-noise ratio (SNR) without advanced data processing at each pixel, a spectrum was averaged 100 times, resulting in a pixel dwell time of 16 ms (or 250 μs per spectral point). Depending on the application and on the degree of data processing, the number of spectral points can be reconfigured dynamically for faster imaging speed. For example, when imaging not in the fingerprint region, the stronger Raman signals permit faster imaging, so Supplementary Fig. 10 is an hyperspectral image (32 spectral points) of polystyrene (PS) and polymethyl methacrylate (PMMA) beads, with 636 μs pixel dwell time (19 μs per spectral point) where pixels were eight times averaged.

In Fig. 4 the hyperspectral TICO-Raman contrast allows for molecular identification with high spatial resolution and high contrast. Figure 4b shows the spectra at pixels P1 and P2. The great advantage of numerous spectral points is evident, as lignin and olive oil can easily be identified and spectral integration over the Raman bands in post processing allows for optimal molecular contrast. The spectral integration regions are marked in green and red. The spectra were Savitzky-Golay filtered and offset corrected by a polynomial fit.The high specificity of TICO-Raman is shown in Fig. 4c–d where each species is displayed individually.

The images in Fig. 4a–d use the SRG signal of the probe beam. In addition, the transmission of the pump beam can generate a transmission microscopy image. Mapping the molecular colour code onto the high definition and high-resolution morphology from the transmission microscopy image then provides the maximum amount of information in a single image (Fig. 4e). This multi-modal image allows to simultaneously identify the sample morphology with very high detail and the molecular composition with functional specificity.

## Discussion

The presented results show the capabilities of the TICO-Raman system. To improve the system sensitivity and with it the imaging speed, it is possible to amplify the FDML Raman probe laser. A 300-times higher power of ∼1.5 W was already demonstrated by using erbium fibre amplifiers^30^ and 75 mW at 1,550 nm (ref. 30) or 100 mW at 1,310 nm (ref. 26) using semiconductor optical amplifiers. The latter option would maintain the broad spectral coverage. Depending on which option will prove most suitable and depending up to which levels shot noise limited sensitivity can be maintained, one can expect a factor 10–100 in signal-to-noise enhancement, that is, a factor 100–10,000 in speed in the future. Another possibility with the flexible TICO-concept is that sparse spectral sampling can further increase the imaging speed (see also Supplementary Fig. 11). This way, one can digitally choose the set of Raman transitions to be recorded and therefore optimize the imaging speed, if the sample molecular composition is known already. Besides the mentioned improvements, the TICO-Raman microscopy system further offers a straight forward extension to future multi-modal endoscopic imaging. Both lasers, TICO-Raman pump and TICO-Raman probe, can also be used for imaging modalities other than Raman. We have already demonstrated that FDML lasers can also be used for record high-speed endoscopic intravascular OCT imaging^27^. In addition, the TICO-Raman pump laser has sufficient power levels for two-photon microscopy and second harmonic imaging. For *in vivo* OCT and two-photon microscopy applications, the long wavelengths of both light sources are very attractive for deep tissue imaging. Together with the robustness of the setup, TICO-Raman is the ideal candidate for molecular-sensitive endoscopy in a real clinical setting.

## Methods

### Experimental setup

Two FDML probe lasers at 1,300 and 1,550 nm are used, both configured to a wavelength sweep repetition rate of either 55 kHz for spectroscopy or 415 kHz for microscopy (see also Supplementary Figs 8,9). The sweep rate had to be reduced in the spectroscopy application due to the 100 MS s^−1^ sampling rate of our current arbitrary waveform generator (AWG). As pump laser, a fibre MOPA was developed to generate arbitrary time encoded pulse patterns synchronized to the FDML sweep in order to cover the whole spectrum of energy differences. The MOPA pump comprises an electronically controllable Raman shifter to select between 1,064 and 1,122 nm pump wavelength. In combination with the two probe lasers, this provides a coverage of Raman transitions from 750 to 3,150 cm^−1^. Both fibre lasers are collimated and combined in free space by dichroic mirrors before being focused on the sample by aspheric lenses (numerical aperture (NA) ∼0.5). After filtering out the pump light by long-pass filters, the probe light is detected on the first port of a homebuilt balanced photoreceiver. On the second port, reference light directly from the FDML laser—without Raman signal—is used for balancing. This first analogue balancing step removes the probe light offset and suppresses intensity noise. Thus, only the SRG signal is acquired with a 1.6 GS s^−1^, 12-bit analogue to digital converter. A second balancing step is performed digitally by exploiting the sweep-to-sweep correlation of the swept probe laser. The shot-noise limited SRG signals are processed to a spectrum in the LabVIEW measurement software. To generate an image, the sample is raster-scanned and a spectrum is recorded at each pixel. For more information, numbers and additional measurements see also the Supplementary Methods and the Supplementary Discussion.

## Additional information

**How to cite this article**: Karpf, S. *et al.* A Time-Encoded Technique for fibre-based hyperspectral broadband stimulated Raman microscopy. *Nat. Commun.* 6:6784 doi: 10.1038/ncomms7784 (2015).

## Supplementary Material

Supplementary Information Supplementary Figures 1-11, Supplementary Discussion, Supplementary Methods and Supplementary References
