# Supplementary material for: A Time-Encoded Technique for fibre-based hyperspectral broadband stimulated Raman microscopy
Source: Nat Commun. 2015 Apr 17;6:6784. doi: 10.1038/ncomms7784 (PMC4410670; doi:10.1038/ncomms7784)
Supplement: Supplementary Information — Supplementary Figures 1-11, Supplementary Discussion, Supplementary Methods and Supplementary References [file ncomms7784-s1.pdf]

Supplementary Figures

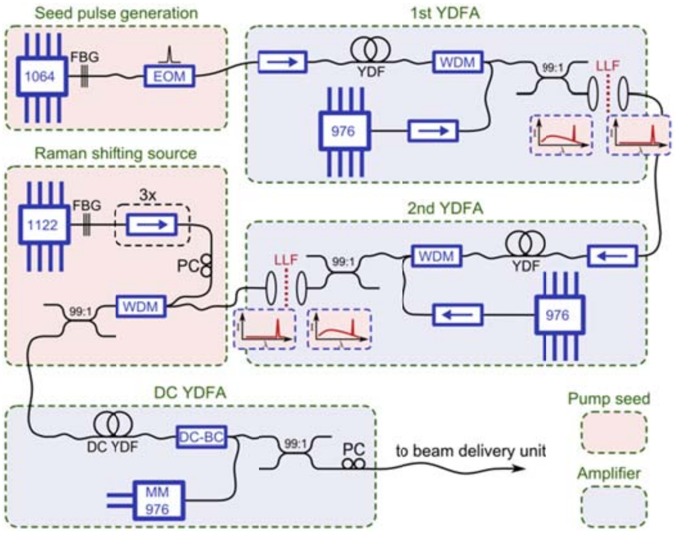

Supplementary Figure 1 Detailed setup of the fibre master oscillator power amplifier (MOPA) pump source. Red highlighted areas mark seed light generation and blue highlighted areas mark amplification stages. In the first seed stage, an electro-optic modulator (EOM) picks short pulses from the narrowband CW 1064 nm seed laser diode, which is equipped with a fibre Bragg grating (FBG). The first two amplification stages are single-mode ytterbium doped fibre amplifiers (YDFA) followed by laser line filters (LLF) while the third stage is a double clad (DC) Yb doped fibre amplifier (DC YDFA). A high power multimode (MM) laser diode is coupled into the DC fibre with a double clad beam combiner (DC-BC). The second seed laser at 1122 nm for Raman shifting is inserted via a wavelength division multiplexer (WDM). The wavelength shifting can be electronically switched on and off by switching the 1122nm diode on and off and adjusting the pump power. Tap couplers (99:1 ratio) are inserted for monitoring purposes. The arrows represent isolators. Polarization is controlled by polarization control paddles (PC).

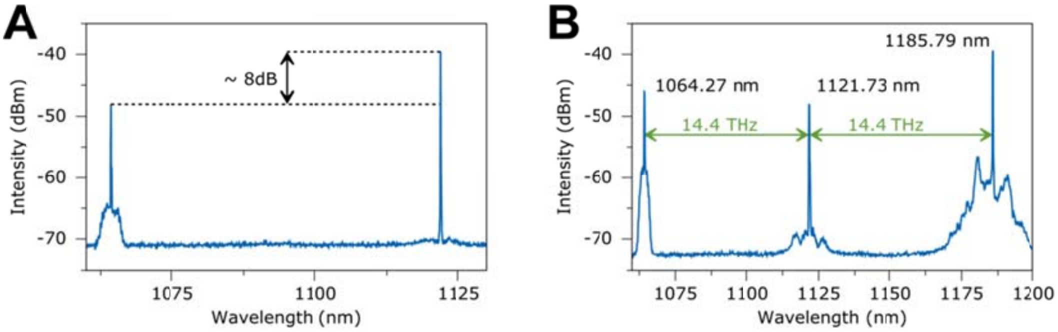

Supplementary Figure 2 Output spectra of the pump pulses. (A) Optical spectrum of the Raman shifted pump pulses. The conversion efficiency from 1064 nm to 1122 nm in the fibre was up to 90% (~8 dB) of the initial pump power. (B) Further cascaded Raman shift in fused silica is achieved when increasing the YDFA pump power.

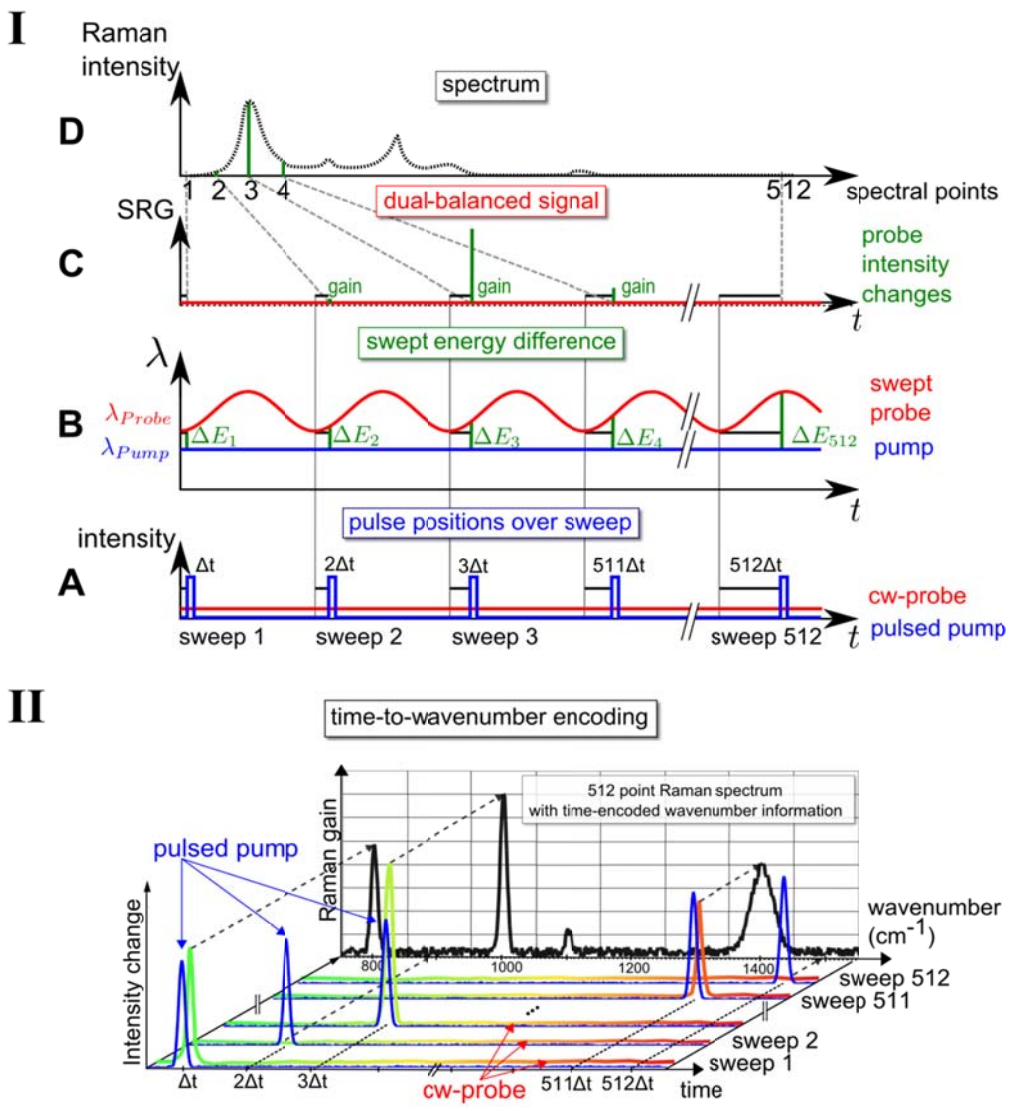

Supplementary Figure 3 Generating stimulated Raman spectra from a swept laser source. (I-A) In order to tune the energy difference between pump and probe lasers, the pump pulses are synchronized to the sweeps of the probe laser in a way, that at every sweep the pump pulses are delayed by a multiple of Δt. (I-B) Each timing corresponds to a distinct energy difference. (I-C) Probe intensity changes due to stimulated Raman gain (SRG) occur whenever the energy difference coincides with a Raman transition. (I-D) Stimulated Raman spectra are recorded by mapping the intensity changes to spectral positions. (II) These spectral points are mapped to wavenumber using the time-to-wavelength relation of the periodically swept probe laser. The intensity change of the probe laser is shown in rainbow colours. The

black spectrum is the final Raman spectrum generated from the individual SRG signals and the time-encoded wavenumber information.

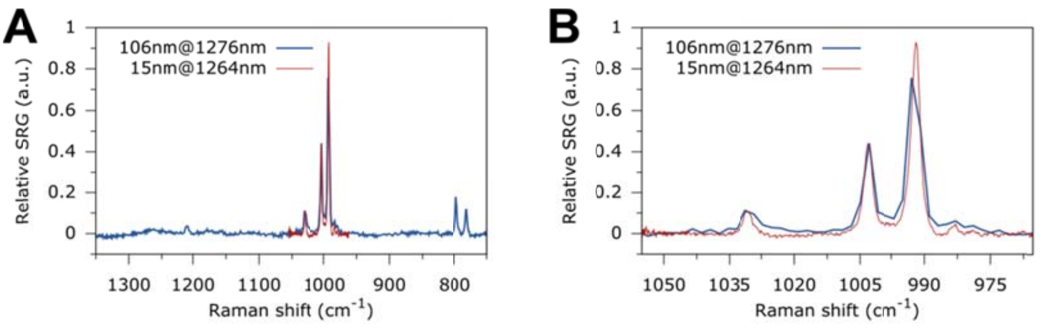

Supplementary Figure 4 Spectral zooming of a liquid mixture. (A) Spectra of a mixture of equal parts of cyclohexane, benzene, and toluene. The blue spectrum was acquired with a wavelength span of 106 nm around a centre wavelength of 1276 nm and the red with 15 nm span around 1264 nm, resulting in a spectral resolution of < 3 cm^{-1} and < 0.5 cm^{-1}, respectively. (B) With spectral zooming, the narrowband peaks are clearly better resolved.

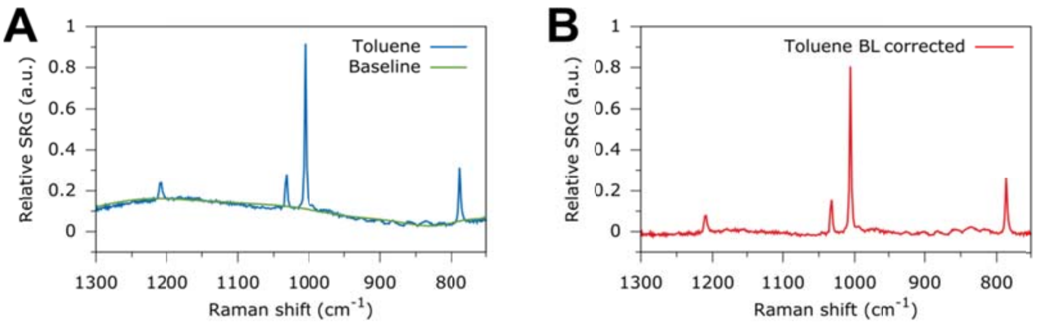

Supplementary Figure 5 Baseline subtraction for broadband spectra. (A) The raw spectrum exhibits a small intensity offset. Therefore, a baseline (BL) was created by an automated algorithm in Origin Software. The generated baselines were then subtracted from the single spectra. (B) The baseline corrected spectrum of toluene around 1000 cm^{-1}.

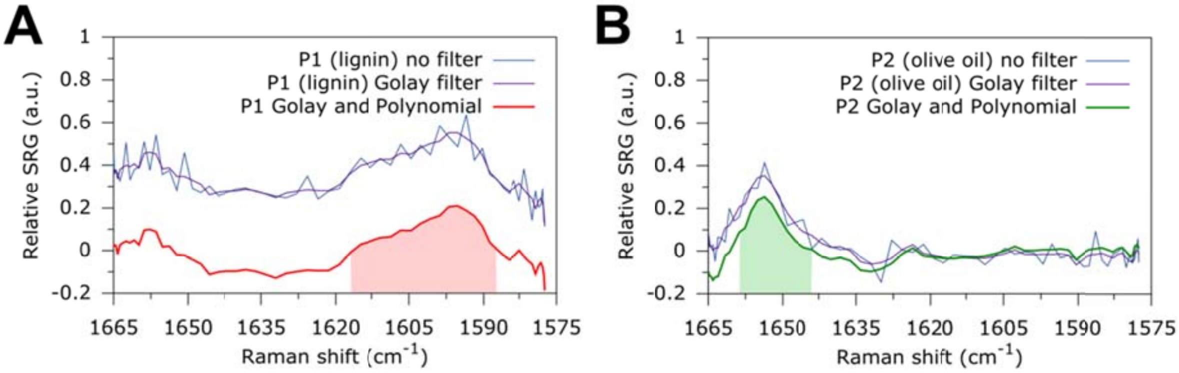

Supplementary Figure 6 Spectra processing for imaging. The 64 point spectra at each pixel were Savitzky-Golay filtered and the offset was removed with a 2nd-order polynomial fit. Figure (A) shows the spectrum of lignin at point P1 (cf. Fig. 4) and figure (B) the spectrum of olive oil at point P2.

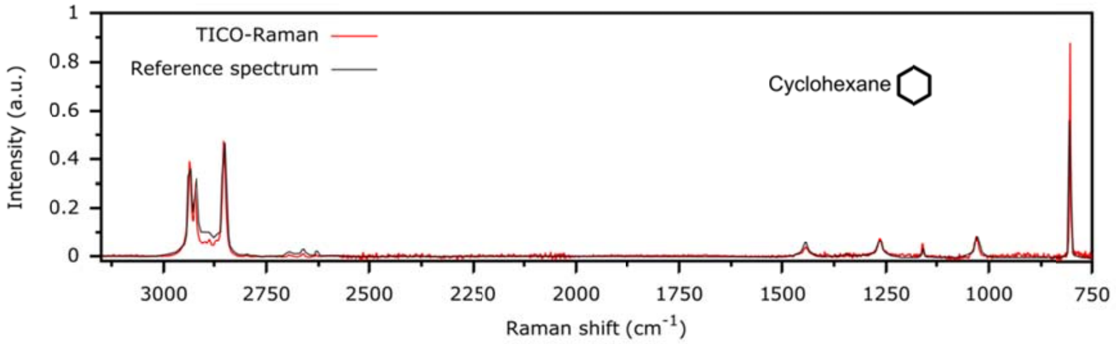

Supplementary Figure 7 Comparison of TICO-Raman spectrum with literature. The spectrum of cyclohexane (red) compared with a spontaneous Raman reference spectrum (black) from the SDBS database [1]. Spectral positions and intensities match very well and the high resolution of our system allows to better resolve the sharp peak of cyclohexane at 802 cm^{-1}.

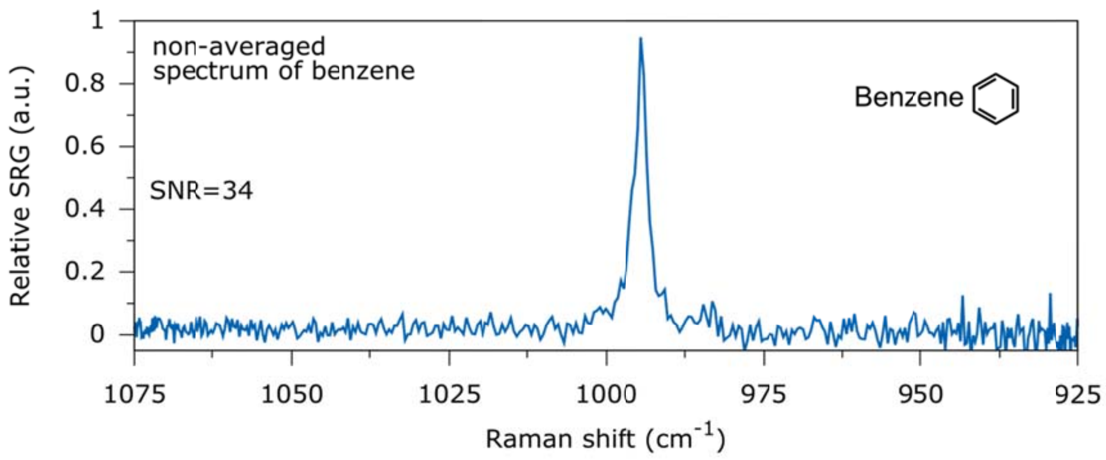

Supplementary Figure 8 Non-averaged TICO-Raman spectrum of benzene. The non-averaged 512 point spectrum was acquired in 9.2 ms using the 55 kHz probe laser and no baseline was subtracted. The signal-to-noise ratio (SNR) of 34 was achieved with an average power of 2.6 mW for the probe and 175 mW for the pump laser.

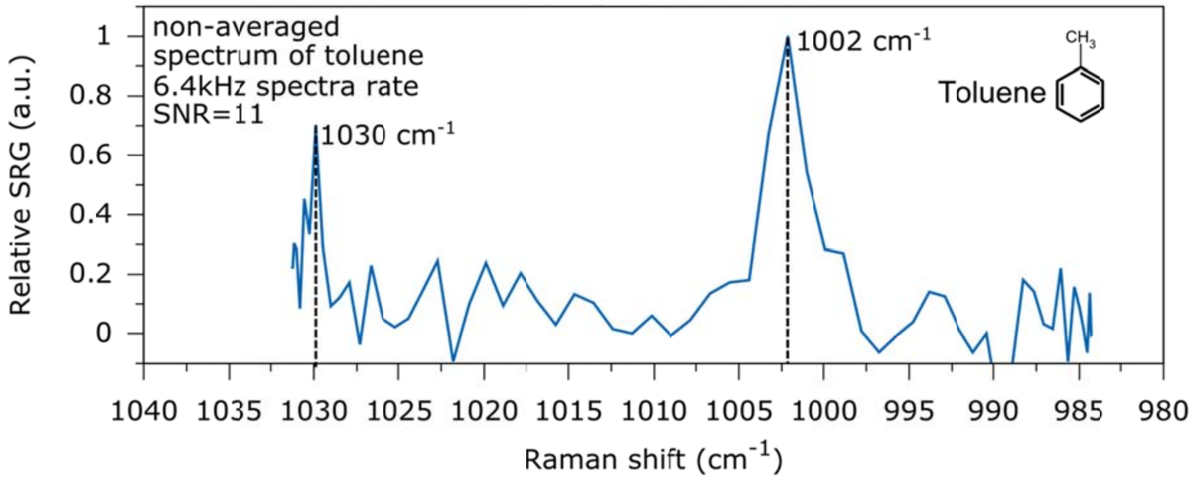

Supplementary Figure 9 Non-averaged TICO-Raman spectrum of toluene. The non-averaged 64 point spectrum was acquired in 157 µs (6.4 kHz spectra rate) using the 415 kHz probe laser and no baseline was subtracted. The signal-to-noise ratio (SNR) of 11 was achieved with an average power of 2.6 mW for the probe and 500 mW for the pump laser.

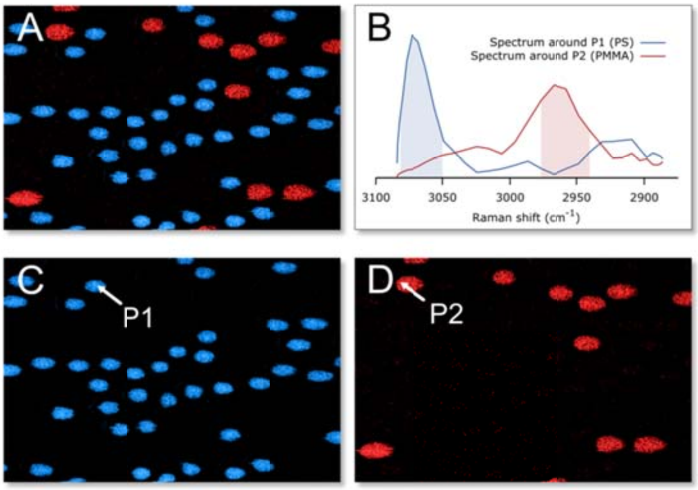

Supplementary Figure 10 Hyperspectral TICO-Raman microscopy image of PS (blue) and PMMA (red) beads. The beads are 20 µm in diameter and dispersed in a water based ultrasound gel. The image size is 480 x 320 pixels (pixel size is 1 µm x 1µm) with 32 spectral points per pixel. A pixel dwell time of 636 µs with the 415 kHz system and 8 times averaging was achieved. (A) PMMA beads are shown in blue and PS beads in red. Their colour channel images are shown in (C) and (D) respectively. The spectra in (B) are Savitzky-Golay filtered and averaged over 5 x 5 pixels. The coloured areas show the spectral points over which the coloured images are averaged for optimal contrast.

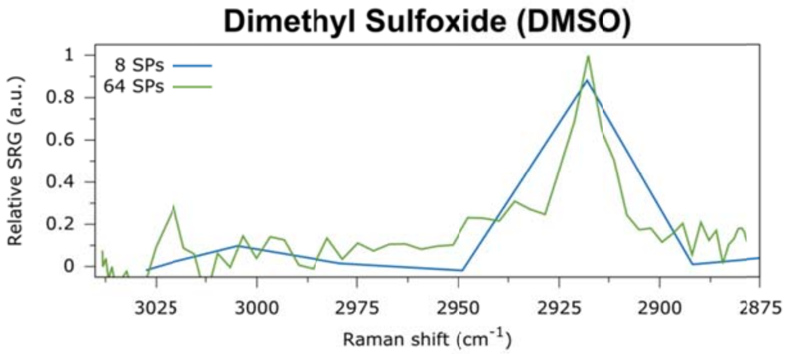

Supplementary Figure 11 Sparse-Sampling: Improved speed for imaging of known Raman transitions. The reduced number of spectral points enables fast acquisition of few Raman transitions. The peak of DMSO around 2915 cm^{-1} can be well resolved with a 64 point

spectrum (green) or faster detected with an 8 point spectrum (blue). The spectra were divided by pump and probe powers and normalized.

Supplementary Discussion

TICO-Raman spectrum compared with literature spectrum

The high quality of the TICO-Raman spectra is best seen when compared with a published reference spectrum given from [1]. The data from [1] was extracted manually and adjusted to a linear x-scale with uniform increments. For the comparison (Supplementary Figure 7), the height of the TICO-Raman spectrum of cyclohexane was scaled to the peak at 1267 cm^{-1} of the reference spectrum, since this peak is least resolution-critical. The spectral positions of all peaks match exactly showing the quality of the time-to-wavenumber mapping of TICO-Raman. Furthermore, the intensities match very well. However, the intensity of the sharp, narrowband peak at 802 cm^{-1} is higher in our spectrum than in the literature spectrum. This is probably because we can better resolve the narrowband peak. The resolution of our system in this region is 1.5 cm^{-1}, limited by sampling density of the broadband sweep. Nevertheless, this resolution is sufficient, as can be seen for a comparable sharp line of benzene at 992 cm^{-1} measured with lower and higher resolution (Fig. 3A).

Fast non-averaged spectrum

The good raw signal-to-noise ratio (SNR) enables acquiring spectra in a fast, non-averaged measurement. This is shown with a 512 point spectrum of benzene at 1000 cm^{-1}(Supplementary Figure 8). The measurement time for the entire spectrum was 9.2 ms, the time per spectral point was 18 µs (acquired with the 55-kHz probe laser). The SNR of 34 in this non averaged acquisition was achieved with an average power of 2.6 mW for the probe and 175 mW for the pump laser. With the 415 kHz System, the measurement time can be reduced down to 2.5 µs per spectral point.

By employing faster FDML lasers and higher pulse repetition rates, we recorded a 64-point spectrum of toluene in 157 µs (Supplementary Figure 9). The 415 kHz FDML allows for these spectra with 6.4 kHz acquisition rate. The SNR of 11 was achieved with an average power of 2.6 mW for the probe and 500 mW for the pump laser (higher than in Supplementary Figure 8 due to the higher repetition rate).

Imaging of polystyrene (PS) and poly methyl methacrylate (PMMA) beads

Supplementary Figure 10 shows a faster hyperspectral TICO-Raman image of a mixture of PS and PMMA beads dispersed in ultrasound gel. The beads are 20 µm in diameter, pixel size is 1 μm^2 and the image size is 480 x 320 pixels. 2 mW of probe power and 400 W of instantaneous pump power (500 mW average power) were used. At each pixel a 32 point spectrum was acquired and averaged 8 times, resulting in a pixel dwell time of 636 µs, or 19 µs/spectral point with the 415 kHz system. The spectra were then Savitzky-Golay filtered using three side points and second order polynomials. Supplementary Figure 10B shows two spectra of PMMA and PS, where 5 x 5 pixels were averaged at the points P1 and P2, respectively. The coloured areas indicate the spectral points over which the coloured images were averaged.

Sparse-Sampling: Specifically tailoring the number of spectral points

For most Raman microscopy applications, more than one spectral point per pixel is desired but less than 100 are required. For most cases 4-20 points will be sufficient to provide enough information for a molecular contrast and at the same time are few enough to still enable high speed imaging. In our TICO-Raman system, the number of spectral points can be adjusted and tailored to the specific application. Thus it is possible to increase the acquisition speed by reducing the spectral sampling if one knows the sample composition beforehand and only certain Raman transitions are of interest. The concept of sparse-sampling is shown here for

dimethyl sulfoxide (DMSO). The Raman transition at 2915 cm-1 can be identified in both the 64 point and the 8 point spectrum (Supplementary Figure 11). The difference in peak intensity is probably due to the fact that, for the 8 point spectrum, the centre wavelength of the probe laser had to be carefully controlled in order to let one spectral point match the narrow Raman band. This may have drifted slightly during the measurement. This may not show up in the wavenumber mapping which was updated just before the measurement. Nevertheless, this shows the concept of sparse-sampling which allows for increasing the acquisition speed if the Raman bands of interest are known in advance.

Supplementary Methods:

Experimental setup FDML Probe laser

The time-encoded Raman (TICO-Raman) spectra are generated by tuning the energy difference between pump and probe lasers. In this paper we used two Fourier Domain Mode locked (FDML) lasers with centre wavelengths around 1300 nm and 1550 nm. Both lasers can be changed from 415 kHz sweep repetition rate to 55 kHz by replacing the tuneable optical filter and the fibre delay loop, which are connected via standard FC/APC fibre connectors. We used the 55 kHz FDML lasers for spectroscopy and the 415 kHz FDML lasers for microscopy to enable faster image acquisition. Two versions are necessary because the product of the driving frequency of the FDML laser and the number of spectral points must not exceed the internal sample clock frequency (100 MHz) of our arbitrary waveform generator (AWG) (TTi, TGA12104). This will be explained in more detail in the paragraph on the spectra generation with the AWG.

The FDML lasers at 55 kHz used fibre Fabry-Pérot tuneable filters (FFP-TF) from Lambda Quest. The 415 kHz systems are based on homebuilt filters. The laser gain elements are fibre coupled semiconductor optical amplifiers (SOA) with a centre wavelength of 1300 nm (Thorlabs, BOA1130S) and 1550 nm (Thorlabs, BOA1104S). The FDML lasers were built with single mode couplers and fibre optical isolators. With an extra cavity booster SOA, average output power levels can be amplified up to 100 mW.

Experimental setup Fibre MOPA Pump laser

The TICO-Raman pump laser is a homebuilt fibre master oscillator power amplifier (MOPA), based on ytterbium doped fibre amplifiers (YDFA). It provides freely programmable pulses that can be synchronized with the swept probe laser. The pulses for this fibre based source have high instantaneous power and switchable emission wavelengths. The primary seed element is a narrowband, single frequency laser diode (Lumics, LU1064M150), providing a continuous wave (CW) output which is amplitude modulated by an electro-optic modulator (EOM, Photline, NIR-MX-LN 10). The extinction ratio of >20 dB was further improved by pre-modulating the laser diode itself with 100 ns gating time, so that depletion of the following YDFAs by leaking seed power is effectively suppressed.

The first two amplifier stages are homebuilt single mode YDFAs, pumped in the backward direction with single mode 976 nm pump diodes (Lumics, LU0980M450) (cf. Supplementary Figure 1). To eliminate the ASE background of the ytterbium fibre (LIEKKI, YB1200-4/125), laser line filters (Thorlabs, FL1064-10) are incorporated in a free space configuration. For wavelength shifting, a second seed light source (Innolume, LD-1122-FBG-400) is optionally coupled into the fibre, protected by optical isolators. The third, double clad (DC) YDFA serves as a power amplifier and consists of a multimode pumped (Lumics, LU0975T090) ytterbium DC fibre (LIEKKI, Yb1200-10/125DC) and a beam combiner (Avensys,

MMC02112CC0). Besides the laser line filters, which can in the future be replaced by a fibre Bragg grating, the whole setup is fusion spliced resulting in a very robust pump laser source.

After every stage, tap couplers (99:1) are inserted to monitor the pulses. The amplified 1064 nm pulses are delivered to the setup in single mode fiber (Corning, SMF28e).

Performance

Our current hardware can achieve pulse durations of 440 ps and higher, repetition rates of 0 – 50 MHz, power levels of 0 – 5 kW, average output powers of 0 – 3 W, duty cycles of 0 – 100 %, emission wavelengths of 1064 nm, 1122 nm, and 1185 nm (Supplementary Figure 2B), and an instantaneous linewidth of less than 1 pm. Not all combinations of these specifications can be realized simultaneously. For example, stimulated Brillouin scattering (SBS) and stimulated Raman scattering (SRS) in the fibre limit the maximum achievable output power. However, in the pump laser SRS is not an unwanted effect, but instead is productively harnessed to generate the shifted pump wavelength. SBS can be suppressed by reducing the pulse length, which, on the other hand, increases the shot noise of the TICO-Raman system due to the required higher electronic bandwidth. For an optimal trade-off we chose a pulse length of 1.8 ns, with a low-pass filtered detection bandwidth of 400 MHz for spectroscopy and 1 ns pulse length and 800 MHz bandwidth for imaging. The measurements of the spectra were conducted with 1600 W instantaneous power for the 1064 nm light and 500 W for 1122 nm light, for the images 720 W pulses at 1064 nm were used. The two pump wavelengths enabled generation of gapless TICO-Raman spectra from 750 cm^{-1} to 3150 cm^{-1}. The pump laser linewidths were below 0.06 nm.

The wavelength shift from 1064 nm to 1122 nm is accomplished by switching on the 1122 nm seed laser diode and increasing the pump power of the DC YDFA stage (cf. Supplementary Figure 1). Hereby, the energy is effectively converted to the longer wavelength within the delivery glass fibre through SRS in fused silica [2, 3]. The high efficiency is shown in Supplementary Figure 2A, where the 1122 nm light exceeds the 1064 nm light by 8 dB. The narrowband linewidth is preserved. With even more power in the DC YDFA stage, it is possible to shift the pump light wavelength even further to ~ 1185 nm (Supplementary Figure 2B). With this 1185 nm pump light, the spectral coverage of the system might be extended down to 250 cm^{-1} which can be important for samples with very low frequency Raman modes in the future.

Noise suppression

The FDML probe laser already provides low intensity noise, only about 20 dB above the shot noise for 1 mW power on the photodiode and a detection bandwidth of 100 MHz. Low intensity noise is a prerequisite for measuring small signal changes on top of a large offset. A further, very unique feature of FDML is the good correlation between consecutive sweeps. Because an FDML laser optically stores the light field of the wavelengths sweep, every sweep is an optical copy of the last one and the remaining noise is strongly correlated. This enables extremely efficient digital referencing algorithms.

A second mechanism to reduce noise is the use of dual balanced, differential detection with a pair of matched photodiodes. For this purpose the probe laser is split into a reference and a sample arm in the beam delivery unit (Fig. 2C). Both signals are detected with a photoreceiver outputting only the difference in the signal. This allows electronic common mode rejection of the probe laser light of up to 40 dB to be achieved. Additionally, the balancing subtracts the probe offset (Fig. 2E) so the depth resolution of the analogue-to-digital converter (ADC) is optimally utilized for signal digitization.

A third mechanism to reduce noise and, more importantly, to reduce artefacts is digitally subtracting signals from two consecutive sweeps of the probe laser, one with and one without pump light. This step eliminates chromatic differences in the splitting ratio between the two optical pathways and interference effects causing spectral ripple and it reduces acoustic and thermo optical crosstalk. This concept also functions as an additional noise filter for the probe laser to further suppress the 1/f-noise.

With the combination of these measures, our system achieves shot-noise limited relative sensitivity of $1.8 \times 10^{-8}\ \frac{1}{\sqrt{\mathrm{Hz}}}$ at 2 mW probe light. With a 1.8 ns gate time, a relative

transmission change of $3.6 \cdot 10^{-4}$ can be measured without averaging. This value was confirmed experimentally.

TICO Modulation pattern for successive spectral sampling

The spectra are generated by scanning the energy difference between pump and probe laser (Supplementary Figure 3). This is achieved by using arbitrary waveforms to drive the whole laser system. The FDML laser is driven by a sine wave where the amplitude determines the span of the sweep and the DC bias voltage controls the centre wavelength. A second synchronized channel drives the electronic pulse board (EPB) to drive the EOM for pump pulse generation. The concept of raster scanning is depicted in Supplementary Figure 3D. At each sweep, the pulse is set at a different relative time position of the sweep, thus corresponding to a different wavelength position of the sweep (cf. Supplementary Figure 3B). Hence, every point in time of the sweep corresponds to a distinct energy difference between pump and probe. With this time-to-wavenumber mapping, the stimulated Raman gain (SRG) signals are used to generate Raman spectra (Supplementary Figure 3A). Furthermore, the SRG on the probe laser is measured, instead of the SRL on the pump, as the signal scales as:

$$\frac{dP_{probe}}{P_{probe}} \propto P_{pump}$$

The analog holds for the SRL on the pump, but as the pump power is about 5 orders of magnitude higher than the probe power, the relative change on the probe is on the order of 10^{-2}, while the relative loss on the pump is only 10^{-7}.

The following parameters were used for the 55 kHz probe lasers for full spectra acquisitions (numbers for 415 kHz lasers used for imaging in brackets). The 4-channel AWG (TTi, TGA12104) can be run at sample clock frequencies up to 100 MHz. For the 55 kHz FDML lasers and 1024 samples per period this corresponds to a sample clock speed of 1024 * 55 kHz = 56.320 MHz. This enables 512 spectral points to be used for one sweep direction, i.e. for half the FDML period (64 for 415 kHz FDML lasers). The pulse pattern increases the pulse position stepwise per sweep period.

As input to the PLL (Analog Devices, EVAL-ADF4350), a sine wave at half the sample clock speed , i.e. (1024*55 kHz)/2=56.320 MHz/2=28.160 MHz (26.560 MHz for 415 kHz lasers) was programmed. The PLL factor was set at 16 times the input frequency (58 for 415 kHz) to produce a sample clock input of approximately 451 MHz (1540 MHz for 415 kHz) for the fast ADC card (AlazarTech, ATS9360).

The ADC card is triggered once for every spectrum, so every 513th FDML period. Although after 512 periods the whole spectrum is already scanned by the pulse, another sweep without pulse is added to the end of one acquisition to serve as background reference. This is due to the pulse height calculation, where we subtract consecutive sweeps for digital balancing. The 513th blank sweep at the end of each waveform provides the subtrahend to the last pump pulse position.

Time-to-wavenumber encoding

The sinusoidally-driven FDML probe laser provides inherent time encoded wavelength information. This is due to the phase-locked, electronically driven filter element. The wavelength sweep can be expressed as:

$$\lambda_{probe}(t) = \lambda_{center} + \frac{\lambda_{span}}{2} \cos\left(2\,\pi \frac{t}{T} + \Phi\right)$$

where T, the inverse of the drive frequency, is the sweep period, λ_{center} and λ_{span} are the center wavelength and the sweep range, respectively. The phase Φ depends on the initial position of the filter and is set to zero on the AWG. One sweep direction is used for spectral acquisition and sampled on the ADC with 512 spectral points. Therefore, the wavelength of a sample $\#S$ is:

$$\lambda_{probe}(\#S) = \lambda_{center} + \frac{\lambda_{span}}{2}\cos\left(\pi\frac{\#S}{512}\right)$$

The Raman transition wavenumber corresponding to the energy difference between pump and probe light for a given sample is given by:

$$\nu(\#S) = \frac{1}{\lambda_{pump}} - \frac{1}{\lambda_{probe}(\#S)}$$

The centre wavelength and the sweep span are recorded with an optical spectrum analyzer (Yokogawa, AQ6370). The sample point array was then computed using a LabVIEW measurement program.

Since the FDML sweeps a cosine function in wavelength, the wavelength difference between consecutive samples varies. At the edges of the sweep range, successive samples have less difference in wavelength than near the centre wavelength. For the resolution of the Raman spectra, the maximum wavenumber step between two adjacent spectral points was taken. This holds as long as the linewidths of the applied lasers do not dominate the resolution. Currently the FDML lasers can provide linewidths better than 50 pm and the pump laser better than 60 pm. This results in a minimal spectral resolution of below 0.5 cm^{-1} for our current setup.

Beam delivery unit

The pump and probe light is delivered to the spectroscopy and microscopy setup in optical single mode fibre. The polarization of both arms is controlled by fibre polarization controller paddles (Thorlabs, FPC560). The outputs of the probe and the pump laser are collimated using aspherical lenses (Thorlabs, A280TM-C) producing collimated beam diameters of ~

3.5 mm. All pump wavelengths exit the same fibre, while the two FDML lasers have two separate collimators. At the pump output, a short-pass filter (Edmund Optics, 84649) is inserted to block the small fraction of cascaded spontaneous Raman light generated in the fibre. If 1122 nm light is used, an additional longpass filter (Edmund Optics, 48565) was inserted to block the remaining 1064 nm light. After passing through a dichroic mirror (Edmund Optics, 84674) to combine the probe with the pump light, the two beams are focused onto the sample with an aspheric lens (Thorlabs, C230TME-C). This generates a focal waist of ~ 1.25 µm for the 1550 nm probe (NA ~ 0.5). The sample was vertically mounted on a three-dimensional translation stage (Thorlabs, PT3-Z8) to enable raster scanning. The scanning is synchronized to the acquisition via LabVIEW software.

After the sample, the beams are recollimated by an aspheric lens (Thorlabs, C230TME-C). The pump light is filtered out by four dichroic filters (2 x Edmund Optics, 84674 and 2 x Thorlabs, FEL1250) to produce > 110 dB suppression. The attenuation of the pump intensity of > 110 dB assures detection of < 10^{-5} probe intensity changes (1 kW pump power compared to ~ 2 mW of probe power).

The transmitted probe light is then focused onto a fast differential photodiode (WieserLabs, WL-BPD1GA). The light from the sample arm is directly focused on an AR-coated photodiode (Fermionics, FD300W) while the light from the reference arm glass fibre is plugged into an FC-mount coupled photodiode (Fermionics, FD300FC). To avoid reflections causing interference patterns, all single mode fibre connectors are angle polished connectors (FC/APC).

Dynamical spectral zooming

Spectral zooming is achieved by reducing the span of the probe FDML laser while keeping the number of spectral samples constant. Spectral zooming allows a narrow region of interest to be sampled more densely, resulting in higher spectral resolution. The region can be chosen

freely within the bandwidth of the probe laser. The spectra in Supplementary Figure 4 were acquired with the same FDML laser. For the overview record in blue, the wavelength span was set to 106 nm around a centre wavelength of 1276 nm. With the 1122 nm pump, this results in a spectral coverage from 736 cm^{-1} to 1388 cm^{-1} with a spectral resolution of < 3 cm^{-1}. The spectrum with higher resolution in red was acquired with a 15 nm span around 1264 nm, thus covering the range from 960 cm^{-1} to 1055 cm^{-1} with a resolution < 0.5 cm^{-1}. In this example, the spectral zooming allows for a more accurate determination of the peak height and the neighbouring peaks of toluene (1005 cm^{-1}) and benzene (992 cm^{-1}) are more readily resolved.

Sample preparation for spectroscopy

For the chemical samples, we used thin strips of 30 µm thick PTFE sheets placed on microscope slides and covered with a No.1 coverslip (~ 160 µm in thickness). The liquid samples could then be deposited on the edge of the coverslip and entered the compartment by capillary forces. To avoid evaporation, the compartments were then sealed with conventional glue which did not enter the sample volume due to its higher viscosity. We used pure liquid chemicals for spectroscopy (SigmaAldrich). The beams enter through the coverslip side to minimize chromatic aberrations introduced by the glass, as the coverslip is thinner than the microscope slide.

Sample preparation for microscopy

For the images of the microbeads (Supplementary Figure 12), we mixed polystyrene (PS) (Microparticles GmbH, PS/Q-F-B1246, 19.67 µm) and poly(methyl methacrylate) (PMMA) beads (Microparticles GmbH, PMMA-F-L621, 21.83 µm) in ultrasound gel to form a monodispersed emulsion. A droplet of the emulsion was placed on a microscope slide,

covered by a coverslip and sealed with glue. For the geranium phaeum image (Fig. 4), we cut about 60 µm thick slices out of the stem with a microtome (Euromex, MT.5503) and placed it on a microscope slide. A droplet of conventional olive oil was added.

Spectra data processing

The spectra of the chemicals were recorded with a 55 kHz sweep rate. Two FDML lasers centred at 1300 nm and 1550 nm can be combined with the 1064 nm or the 1122 nm pump, resulting in a total coverage from 750 cm^{-1} to 3150 cm^{-1}. The acquisition time of a single raw 512 point spectrum was 9.2 ms (time = $[\text{FDML laser frequency} / (\text{no of spectral points}+1)]^{-1}$). The spectra were averaged 1,000 times for a high signal to noise ratio. Since the system operates at the shot noise limit, more averaging improves the quality of the spectra even further.

A small remaining offset was compensated by an automated baseline subtraction in Origin Software (Supplementary Figure 5). No subtraction was performed for the linearity measurement of the chemical mixture (Fig. 2B,C) and the non-averaged spectra (Supplementary Figure 8,9). For the broadband spectrum (Fig. 3), the four spectra were merged to 1565 spectral points, since redundant points in the overlap regions were omitted.

Image data processing– molecular contrasting

The TICO-Raman microscopy images were acquired by raster scanning the sample. The resolution was set to 600 x 400 pixels with 1 µm x 1 µm pixel spacing. At each point a 64-point spectrum was acquired. The 100-times averaged spectra were Savitzky-Golay filtered in 2nd - order with 3 neighbouring points. A remaining offset was corrected with a 2nd - order polynomial fit (cp. fig S6). In post-processing the optimal signal-to-noise ratio was achieved by averaging over 11 neighbouring spectral points of the broad Raman transitions of olive oil and 17 spectral points for lignin (coloured areas in Supplementary Figure 6). The obtained intensity distributions were then exported to an 8-bit grey scale image. After applying appropriate intensity cut levels and rescaling to 8-bit, the images were coloured in an image processing program (Gimp 2) and overlaid by adding the single coloured layers to an RGB image.

Since the sample was removed between the measurements of the Raman contrast and the transmission microscopy image, the images had to be aligned and slightly resized before image fusion (Fig. 4E). There are some partially black lines visible in the image, the origin of these small artefacts is not known.

Supplementary References:

1. SDBSWeb: http://sdbs.riodb.aist.go.jp. (National Institute of Advanced Industrial Science and Technology, accessed 03.03.2014).

2. Stolen RH, Ippen EP. Raman gain in glass optical waveguides. *Applied Physics Letters* **22**, 276-278 (1973).

3. Feng Y, Taylor LR, Calia DB. 150 W highly-efficient Raman fiber laser. *Opt Express* **17**, 23678-23683 (2009).
